# Excited-State Dynamics Leading Either to Triplet Formation or Coordinative Expansion following Photolysis of Cu(II)-Porphyrins: A DFT, TD-DFT, Luminescence and Femtosecond Time-Resolved Absorbance Study

**DOI:** 10.3390/molecules28176310

**Published:** 2023-08-29

**Authors:** Ross J. McGarry, Lazaros Varvarezos, Mary T. Pryce, Conor Long

**Affiliations:** 1 School of Chemical Sciences, Dublin City University, D09 V209 Dublin, Ireland; ross.mcgarry6@mail.dcu.ie (R.J.M.); mary.pryce@dcu.ie (M.T.P.); 2 School of Physical Sciences, Dublin City University, D09 V209 Dublin, Ireland; varvarezos.lazaros@gmail.com

**Keywords:** porphyrin, copper, photophysics, DFT, TD-DFT, luminescence, transient absorption, femtosecond

## Abstract

The photophysical properties of Cu(II) complexes with 5,10,15,20-*meso*-*tetrakis*(phenyl)porphyrin and 5,10,15,20-*meso*-*tetrakis*(*N*-methylpyridium-4-yl)porphyrin are examined via the luminescence and femtosecond time-resolved absorbance methods, respectively. These studies are supported by DFT and TD-DFT calculations, which highlight the important role played by ligand-to-metal charge-transfer states in directing the system toward either intersystem crossing to the triplet hypersurface or coordinative expansion to a five-coordinate quasi-stable intermediate. The latter processes occur when the porphyrin is photolyzed in the presence of suitably located Lewis bases. Femtosecond time-resolved absorbance measurements of Cu(II)-5,10,15,20-*meso*-*tetrakis*(*N*-methylpyridium-4-yl)porphyrin confirm that the coordinative expansion in water occurs in approximately 700 fs, while crossing to the triplet hypersurface takes approximately 140 fs in the same solvent. These processes are mutually exclusive, although both can occur simultaneously depending on the environment of the porphyrin. The ratio of the two processes depends on the relative orientation of the Lewis base with respect to the copper atom at the time of excitation. As a consequence, copper porphyrins such as these are excellent probes in the environment of the porphyrin and can be used to identify the location of the porphyrin when interacting with DNA fragments.

## 1. Introduction

**Figure 1:**
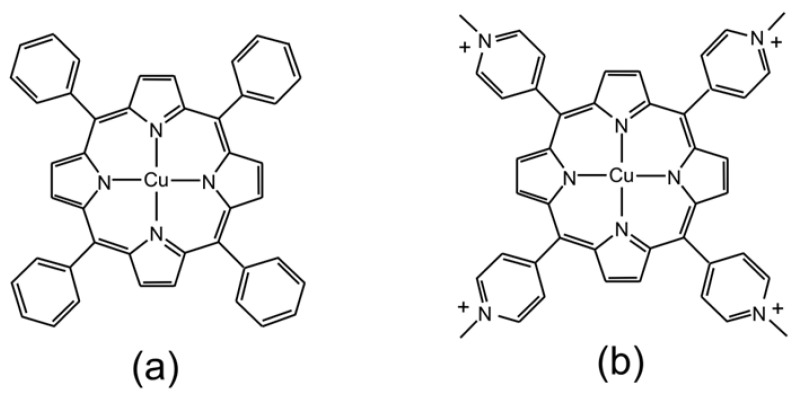
Representations of the molecular structures of (**a**) CuPh and (**b**) CuPy.

There are numerous papers describing the photophysics and photochemistry of porphyrins with a wide variety of *meso*-substituents and in a wide range of solvent systems [1,2,3,4,5,6,7,8,9,10,11,12]. Copper-containing porphyrins are particularly interesting, with complex photophysical properties, and they are amongst the most widely studied of the paramagnetic metalloporphyrins [13,14,15,16,17,18,19,20,21,22,23,24,25,26,27,28,29,30,31,32,33,34,35,36,37,38,39,40,41,42,43,44,45,46,47]. Given the broad range of systems studied, it is difficult to assign specific photophysical properties to specific substituent or solvent selections. As a result, we have decided to limit our investigations to two porphyrins: first, the copper complex with 5,10,15,20-*meso*-*tetrakis*(phenyl)porphyrin (Figure 1a; CuPh) and, second, the copper complex with 5,10,15,20-*meso*-*tetrakis*(*N*-methylpyridium-4-yl)porphyrin (Figure 1b; CuPy). CuPh is soluble in non-polar organic solvents such as toluene, while CuPy, as its chloride salt, is soluble in water. We have also limited our investigations to these two solvents.

**Figure 2:**
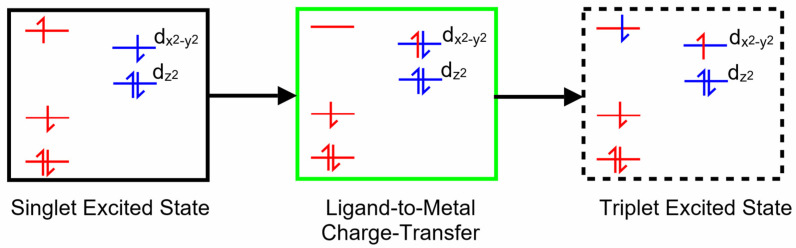
A schematic representation of a singlet excited state of a copper porphyrin (black box), the ligand-to-metal charge-transfer state (green box) and a triplet excited state (dashed box); red levels correspond to molecular orbitals located on the porphyrin with their electrons and blue levels represent orbitals on the copper atom with their electrons.

Copper porphyrins can exhibit characteristics of both porphyrin-centered and metal-centered excited states, and the coupling of porphyrin-based excited states to the unpaired electron on the copper can, in specific circumstances, open an efficient spin-allowed route to the triplet hypersurface. This process is known as enhanced intersystem crossing (EISC) [48], and it is represented schematically in Figure 2. Orbitals and electrons centered on the porphyrin are colored red, while those on the copper atom are colored blue. Of particular interest here is the role of the ligand-to-metal charge-transfer (LMCT) state (green box) in facilitating a spin-allowed route to the triplet hypersurface (dashed box).

The efficiency and ultrafast nature of the EISC process has led researchers to assume that the photophysics of copper porphyrins is dominated by processes on the triplet hypersurface [49]. This includes the weak emission from CuPh in toluene solution, which has been assigned to triplet states that are quenched by the addition of Lewis bases such as pyridine [49]. This was explained by proposing the formation of a five-coordinate complex via the reaction of the triplet excited state(s) with a molecule of pyridine forming an exciplex, i.e., a complex that exists in an electronic excited state but not in the ground state [33,45,49,50,51,52].

Cationic water-soluble copper porphyrins such as CuPy have also been reported to form exciplexes with DNA bases, and for this reason, they have been used to probe a variety of DNA environments [16,17,18,19,33,39,53,54,55,56]. Studies in water usually use Raman spectroscopy, and the expansion of the copper coordination sphere from four to five results in spectroscopic changes associated with porphyrin ring modes in the 1300–1700 Δcm^−1^ range. Unfortunately, Raman spectroscopy is insensitive to the nature of the fifth or axial ligand, and it is only possible to distinguish between different axial ligands by measuring the lifetime of the five-coordinate species. Luminescence techniques are of limited use in studying the photophysics of CuPy as it is non-emissive in water. However, emission is observed in the presence of some DNA fragments, particularly those containing guanine–cytosine base pairs such as the oligonucleotide d(GC)_5_ [57]. It is assumed that the CuPy intercalates between the base pairs of the d(GC)_5_ oligomer, and this prevents interaction between the copper atom and water molecules. This has the effect of “turning on” emission from, it is assumed, a triplet excited state, although the emission lifetime of 20 ns is rather short for phosphorescence.

In this work, the results of luminescence studies of CuPh in toluene are presented, which are supported by DFT and TD-DFT calculations. In the case of CuPy, femtosecond TA experiments were performed either in pure water or in water containing d(GC)_5_. In the first case, water can act both as a solvent medium and also as a potential axial ligand. In the second, water acts simply as a solvent and cannot act as an axial ligand. This allows the excited-state development to be monitored in an aqueous environment in conditions where water has access to the copper atom and where it does not. DFT and TD-TDF calculations are then used to explain the experimental results.

These experiments were designed to answer several important questions. Firstly, it is unclear which excited state or states react with the Lewis base to form the five-coordinate species. Most reports have assumed that it is the low-lying triplet states that are involved, although recent DFT studies have cast some doubt on this [39], as does the observation that the triplet state(s) can still be detected long after all the five-coordinate species have decayed [41]. Previous studies have attempted to provide plausible explanations for this particular observation, albeit with limited success [16,41]. Secondly, there appears to be no plausible explanation as to why the lifetime of different five-coordinate complexes depends so markedly on the nature of the axial ligand. The complex with water has a lifetime of about 30 ps while the thymine complex survives into the nanosecond domain, despite both complexes involving oxygen donor atoms.

In this paper, the quantum chemical approach taken is to systematically investigate the effects of the location of the Lewis bases close to the copper atom on excited-state development in CuPh and CuPy (the optimized coordinates for both porphyrins are presented in the Supplementary Materials in Table S1 and Table S2, respectively). Although this is a very computationally expensive approach, it has the advantage that it does not rely on previous assumptions as to the multiplicity of the reactive excited state(s). The results of these calculations are then compared to the experimental results. This provides a clearer picture of the photophysics and photochemistry of copper porphyrins in general, and it helps answer many of the outstanding questions that impact their use as probes in biological systems.

## 2. Results

**Figure 3:**
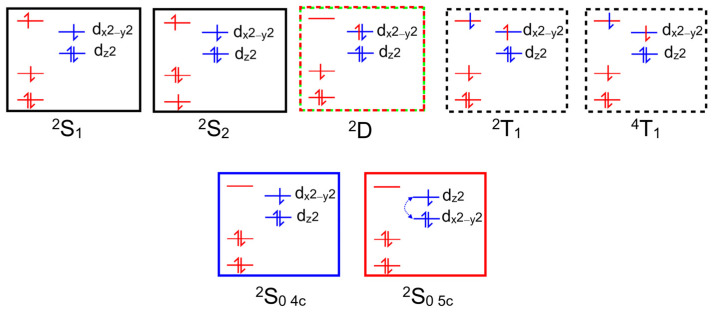
Schematic representations of the optically accessible states of copper porphyrins using the state assignments of Gouterman; the red levels/electrons are associated initially with the porphyrin, while the blue are associated with the copper atom, black boxes indicate S states, red/green dashed D (ligand-to-metal charge-transfer state), black dashed T states, and blue and red indicate ground states.

Copper II complexes have an unpaired d-electron (3d^9^4s^0^), which in the four-coordinate ground state is presumed to be in the d_x_^2^_−y_^2^ orbital. This d-configuration facilitates the location of the copper atom in the plane of the porphyrin ring, while the fully occupied d_z_^2^ orbital inhibits axial coordination of further ligands. Optical excitation can populate either the S_1_ or S_2_ porphyrin-centered excited states. The overall multiplicity of the copper porphyrin is that of a doublet, and Gouterman developed specific terminology to describe this electronic configuration or metalloporphyrins such as these [14]. A superscript numeral represents the overall multiplicity of the metal porphyrin complex, 2 for a doublet, as is the case for the copper porphyrins, while the multiplicity of the porphyrin is indicated by an uppercase letter (S for singlet, D for doublet or T for triplet). The ordering of the states in terms of energy is represented by numeric subscripts. We have added a slight refinement by adding a subscript 4c or 5c to represent four-coordinate or five-coordinate complexes, respectively. Schematic representations of these states are presented in Figure 3, which can act as a reference for the terminology used throughout this paper.

### 2.1. The Photophysical Properties of CuPh in Toluene

#### 2.1.1. The Luminescence Studies

**Figure 4:**
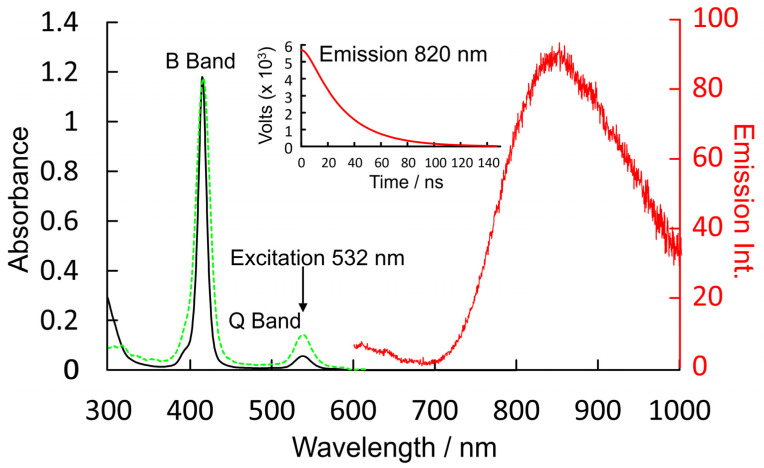
The UV/vis. absorption spectrum (black) showing the B and Q bands of CuPh in toluene, the emission (red) following 532 nm excitation and the time profile data for this emission as the inset; the dashed green spectrum is the excitation spectrum for the emission at 820 nm.

Outlined in Figure 4 are the absorption and emission data for CuPh in toluene solution at room temperature. The absorption spectrum is typical of this class of complex, showing a sharp and intense B-band absorption at 416 nm (ε = 2.796 × 10^5^ m^−1^dm^3^cm^−1^) and a weaker Q-band feature at 540 nm (1.25 × 10^4^ m^−1^dm^3^cm^−1^). Excitation into either band results in a weak emission centered at 820 nm. The lifetime of this emission following 532 nm excitation is 30 ns. This emission lifetime was the same in oxygenated solvent as in degassed (freeze–pump–thaw) solution. The excitation spectrum for this emission has a profile, within the experimental error, identical to the absorption spectrum (green dashed plot in Figure 4).

#### 2.1.2. DFT and TD-DFT Calculations for CuPh in Toluene

**Figure 5:**
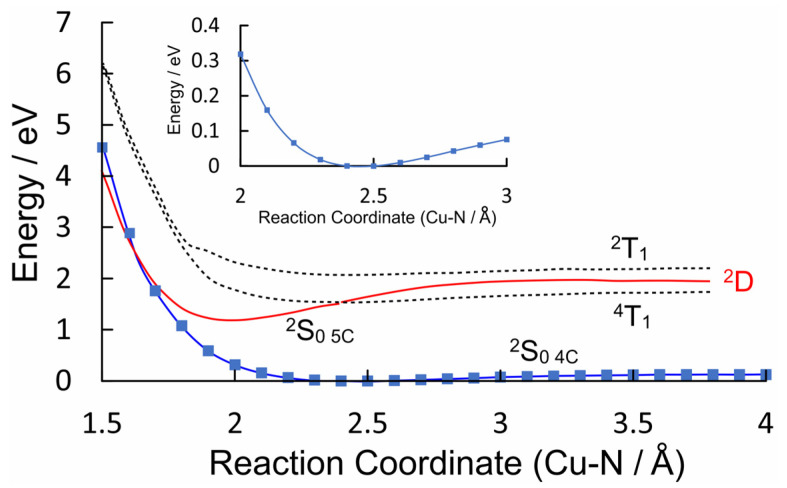
The potential energy profiles of the four-coordinate ground-state of CuPh (^2^S_0_ **_4C_**) as modelled in toluene versus the Cu–*N*(pyridine) distance (Å) (blue plot with data points indicated by blue squares); the ^2^T_1_ and ^4^T_1_ states are indicated by the dashed curves while the ^2^D_0_ state is in red, which develops into the ^2^S_0 **5C**_ at short Cu–*N* distances.

The valence orbital energies and fragment compositions for CuPh are presented in Table S3 and a summary of the electronic transitions is available in Table S4 in the Supplementary Materials. A relaxed potential energy plot was constructed via DFT methods, in which the location of a nitrogen atom of pyridine was held at fixed distances from the copper atom in CuPh. All the other structural parameters were allowed to optimize in these calculations. The resulting plot of energy against the Cu–*N*(pyridine) distances is presented in Figure 5 as the blue plot, with the data points indicated by blue squares. This profile exhibits a small minimum at a Cu–*N*(pyridine) distance of 2.5 Å, which indicates very weak interaction between the copper porphyrin and pyridine. This weak interaction (see the inset in Figure 5) results in a redshift of the B-band maximum [58], and it will affect the time-averaged structure of the CuPh–pyridine complex in the solution. It is estimated that between 35 and 55% of CuPh exists as the five-coordinate complex in neat pyridine at room temperature [50]. 

**Figure 6:**
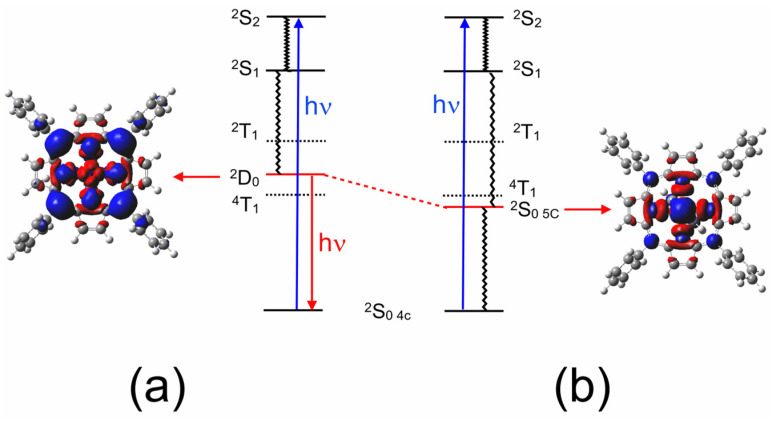
The development of the LMCT (^2^D_0_) state in CuPh as the Cu to *N*(pyridine) distance is reduced from (**a**) 3.5 to (**b**) 1.9 Å, showing how it develops into a metal-centered state (the pyridine molecule is located on the far side of the porphyrin ring so that the electron density changes in the copper atom can be viewed clearly); the blue volumes indicate the regions where the electron density is less in the ^2^D or ^2^S_0 **5c**_ state compared to the ^2^S_0 **4c**_ state and the red volumes indicate the regions where the electron density increases (both electron density maps were cut at the same isovalue of 0.0004).

The ground-state coordinates obtained via the relaxed potential energy calculations were then used to estimate the energies of the excited states of all the multiplicities (the unrestricted functional was used in these TD-DFT calculations) at all points along the reaction coordinate. These energies were then plotted against the Cu–*N*(pyridine) distance. Only the three lowest energy states are presented in Figure 5 for clarity (plots of the 20 lowest energy excited states are presented in the Supplementary Materials in Figure S1). It is clear from these that neither the ^2^T_1_ nor the ^4^T_1_ states (dashed plots) interact significantly with pyridine. Of particular importance to this work is the relative energy of the ligand-to-metal charge-transfer state (^2^D) (red curve) with respect to the triplet excited states (electron density difference maps for the ^2^D, ^2^S_1,_ and ^2^S_2_ states are given in Figure S1, S3–S5, respectively). At all the Cu–*N*(pyridine) distances, the ^2^D state has lower energy than the ^2^T_1_ state. It is clear from the work of Shafizadeh et al. [59] that the ^2^D state is pivotal in determining if an efficient spin-allowed route exists for EISC to the triplet hypersurface. According to the calculations presented in Figure 5, crossing to the triplet surface would be energetically unfavorable and would therefore be, at best, a slow process for CuPh. It is also notable how the character of the ^2^D state changes from LMCT to one that is more metal-centered when the Cu to *N*(pyridine) distance is short (Figure 6). The electron density difference map on the left of Figure 6, i.e., where pyridine nitrogen is remote from the Cu atom, shows large blue volumes on the *meso*-carbons. This indicates that these carbons are involved substantially in the LMCT state. Any change in the nature of the substituents on these *meso*-carbon atoms will, therefore, affect the energy of the ^2^D state and consequently affect the photophysics of the porphyrin. If the Cu–*N*(pyridine) is short, the state is predominantly metal-centered, and blue volumes are now evident on the copper atom, indicating a significant d–d character in this state. This state then develops into the ground state of the five-coordinate species ^2^S_0 **5c**_. As indicated in Figure 5, this ^2^S_0 **5c**_ state crosses to the ^2^S_0 **4c**_ at very short Cu–*N*(pyridine) distances, providing a rapid non-radiative return route to the four-coordinate complex via thermal ligand loss.

### 2.2. The Photophysical Properties of CuPy in Water

#### 2.2.1. The UV/Vis. Absorption of CuPy in Water

**Figure 7:**
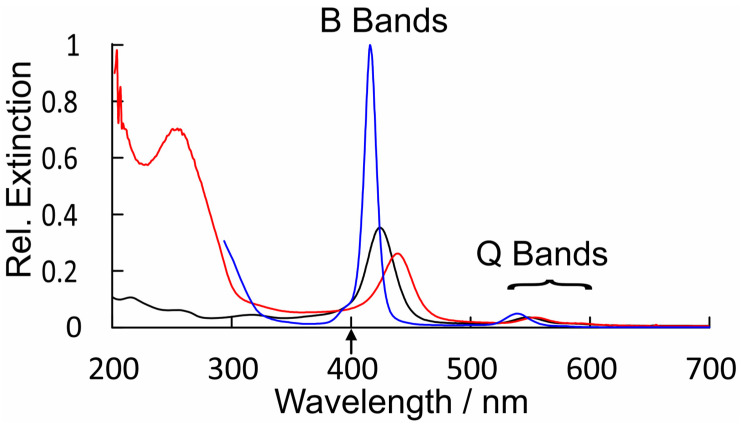
A comparison of the relative extinctions of CuPh in toluene (blue), CuPy in water (black), and CuPy plus d(GC)_5_ in water (red), showing the broadening and redshifts of both the B bands and Q bands for CuPy in water and more particularly in water plus d(GC)_5_; upward arrow indicates the fs-TA pump wavelength.

The UV/vis. spectra of CuPy in water and water plus d(GC)_5_ are presented in Figure 7, along with that of CuPh in toluene for comparison. This spectrum of CuPy is similar to that of CuPh, except the absorption bands are broader, shifted to the red and much weaker. The B-band maximum is at 424 nm (ε = 9.5 × 10^4^ m^−1^dm^3^cm^−1^) compared to 416 nm for CuPh in toluene. This shift does have consequences for the fs-TA studies presented here, which use the second harmonic (400 nm) of the Ti:sapphire fundamental as the pump pulse (upward arrow in Figure 7). This means that the excitation is to the high-energy side of the B-band absorption where the CuPy has a relatively low extinction. For a given porphyrin, for example, CuPh, the redshift of the B band results from changes in the porphyrin environment and can indicate the coordination of a Lewis base to the copper center in the ground state. Such shifts have been used to estimate the equilibrium constants for these interactions [50]. Equally important for this work is the fact that the nature of the transition responsible for the B band changes in moving from CuPh to CuPy. For CuPh, the transition is mainly a porphyrin ring π-π* in character with some metal-to-ligand charge-transfer (MLCT) state character (see Figure S4. For CuPy, however, the B-band transition has substantial intra-porphyrin character, involving porphyrin ring-to-pyridinium charge transfer (Figure S5). Such a transition would be very sensitive to the polarity of the medium. This highlights the influence *meso*-substituents can exert on the photophysics of these porphyrins.

#### 2.2.2. DFT and TD-DFT Calculations for CuPy in Water

**Figure 8:**
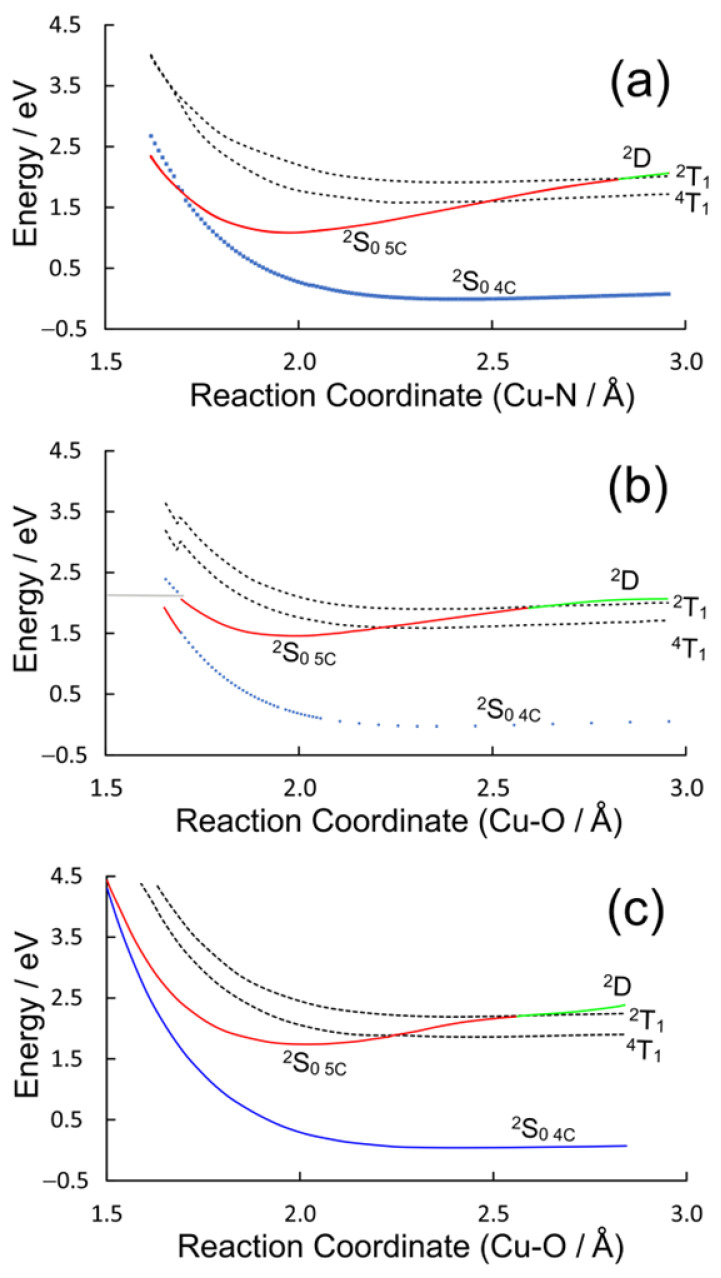
The potential energy plots obtained by varying the Cu–donor atom distance between 1.5 and 3 Å for (**a**) pyridine, (**b**) water, and (**c**) thymine; the blue plots describe the behavior of the four-coordinate ^2^S_0_ ground state, the dashed curves represent the triplet ^2^T_1_ and ^4^T_1_ states and the green curve is the ^2^D state, which develops into the five-coordinate (5c) ground state (^2^S_0 **5c**_ red curve) at shorter Cu–donor atom distances.

The valence orbital energies and fragment compositions for CuPy are presented in Table S5 and a summary of the electronic transitions is available in Table S6 in the Supplementary Materials. Using the same approach as that for CuPh, relaxed potential energy profiles were constructed to model the excited-state development of CuPy in water and in the presence of three Lewis bases: pyridine, water, or thymine. Pyridine was chosen to allow for a direct comparison with the results for the CuPh system described in Section 2.1.2, notwithstanding the fact that experimental verification of these results would be impossible because of solubility issues. Water and thymine were chosen because of reports in the literature that five-coordinate species form with these Lewis bases, although they have very different lifetimes, 30 ps for water while the thymine complex survives for many nanoseconds [17]. The potential energy plots for the three Lewis bases are presented in Figure 8.

The ground-state potential energy curves are weakly binding in all three cases, which indicates a very weak interaction between the copper atom and the Lewis bases. These plots present a broadly similar picture for the dependence of the excited-state energies with the reaction coordinate, although there are some subtle differences (see Figure S2). In all cases, the triplet states (^2^T_1_ or ^4^T_1_, dashed plots) do not interact significantly with the Lewis base. This is consistent with earlier DFT calculations that failed to detect any significant interactions between the triplet excited states and an *O*-donor Lewis base [39]. Their energy profiles broadly follow those of the ground state. Only one state (^2^S_0_ **_5C_**, red plot) presents a substantial energy minimum along the reaction coordinate for each Lewis base, and at the energy minimum, it has the properties of a ground-state species rather than an excited state. If it were a ^2^S excited state, it should be accompanied by a lower energy triplet state. No such state is evident from these calculations. For two of the three Lewis bases, pyridine or water, this ^2^S_0 **5c**_ state crosses the four-coordinate ^2^S_0 **4c**_ ground state at short Cu–donor distances. This provides a facile route to relaxation to the pre-irradiated state. In the case of thymine, however, the ^2^S_0 **5c**_ complex is stabilized by an additional hydrogen bonding interaction between a porphyrin-ring nitrogen and an endocyclic hydrogen of the thymine, and in this case, the ^2^S_0 **5c**_ does not cross to the ^2^S_0 **4c**_ state within the span of this reaction coordinate (see Tables S7 and S8). Consequently, no facile route to ligand loss exists and the five-coordinate species will have a longer lifetime as measured by the time-resolved Raman studies mentioned earlier [17].

### 2.3. Femtosecond Time-Resolved Absorbance Studies of CuPy in Aqueous Environments

The TA experiments described here can be divided into two classes. In the first, water acts as more than just a solvent; it also acts as a Lewis base toward the copper center. In the second, water simply acts as the solvent, and d(GC)_5_ is added to prevent water acting as a Lewis base as the copper porphyrin is intercalated between the GC base pairs and held in a hydrophobic environment [60]. In this way, it is possible to highlight the effect that *meso*-pyridinium-4-yl substituents, as opposed to phenyl substituents, have on the excited-state formation and dynamics. Experiments in pure water highlight the additional role of water as a Lewis base.

#### 2.3.1. Femtosecond Time-Resolved Absorbance Studies of CuPy in Water

**Figure 9:**
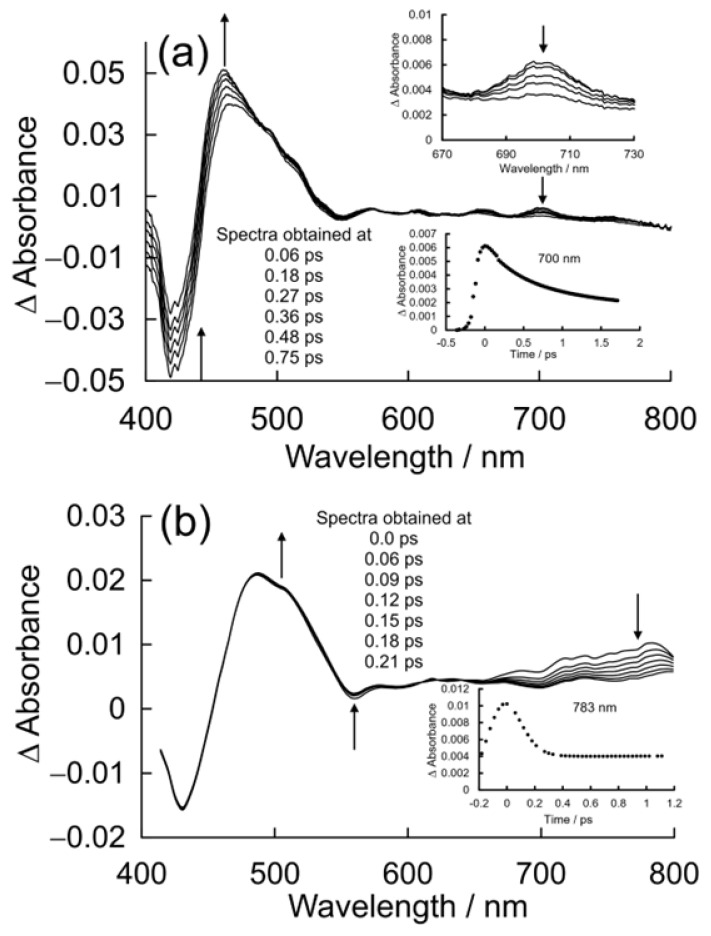
(**a**) The absorbance changes observed following 400 nm pulsed excitation of CuPy in pure water, showing changes consistent with the formation of a five-coordinate species in 700 fs, where the inset shows the temporal evolution of the weak feature at 700 nm; and (**b**) the absorbance changes observed following 400 nm pulsed excitation of CuPy in water with added d(GC)_5_, showing changes consistent with the formation of a triplet excited state and a precursor state with a broad absorbance between 700 and 800 nm, which decays in 140 fs (see inset).

The UV/vis. changes observed following pulsed photolysis (λ_exc._ = 400 nm) of a 0.05 mM solution of CuPy in pure water are presented in Figure 9a. These are difference spectra, where the negative absorbance represents the bleaching of the parent absorptions upon excitation, for instance, the B-band features at 424 nm and the weak Q band at 548 nm. An intense product band is formed within the excitation pulse at ca. 468 nm with very weak features in the visible region, the most intense of which is at 700 nm (see inset). Over the initial 700 fs, this feature decays and the absorbance at 460 nm becomes more intense and narrows. The parent bleach also appears to recover. This recovery signal is deceptive, however, as it corresponds to the development of the overlapping absorbance at 460 nm rather than the reformation of the parent complex. The product absorption occurs immediately to the red of the ground-state bleach and results in a so-called derivative-like difference spectrum, which is characteristic of the formation of a five-coordinate species [61].

#### 2.3.2. Femtosecond Time-Resolved Absorbance Studies of CuPy in Water plus d(GC)_5_

The UV/vis. changes observed following pulsed photolysis (λ_exc._ = 400 nm) of a 0.05 mM solution of CuPy in a buffered (pH 7.0) aqueous solution containing 0.1 mM d(GC)_5_ are presented in Figure 9b. The parent bleach is observed at 430 nm, and it is important to note that this bleach does not recover in the timescale of this experiment (2.1 ps). A broad product absorption is evident at 485 nm, which changes little. Those minor changes are highlighted with arrows in Figure 9b. However, a significant and broad absorption was observed in the region from 650 to 800 nm. This region is known to provide more diagnostic information on the nature of the excited-state species in metalloporphyrin systems [61]. The features in the 400–600 nm region are insensitive to the nature of the excited states involved. The broad absorption in the red region (monitored at 783 nm) decays on an ultrafast timescale with a lifetime of approximately 140 fs, close to the response time of the experimental setup estimated to be 90 fs. No recovery of the parent bleach was observed in this timescale. The product absorbance in the 400 to 600 nm region therefore represents the sequential formation of the ^2^S, ^2^D and ^2^T excited states, all of which have similar absorbances in this region [16,61]. The ultimate product is the ^2^T_1_ state, which is thought to establish an equilibrium with the ^4^T_1_ state [50].

## 3. Discussion

**Figure 10:**
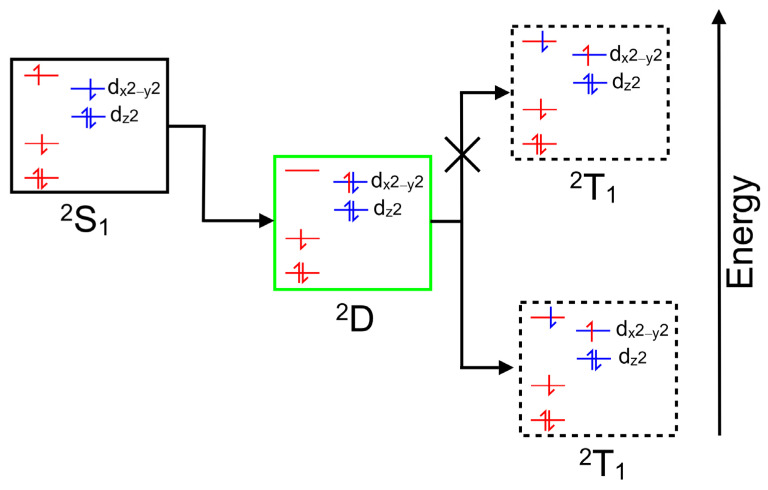
A schematic representation of the EISC to the ^2^T_1_ state, where the ^2^T_1_ energy is less than that of the ^2^D state, while no such crossing occurs if the ^2^T_1_ energy is greater than the ^2^D energy.

The elegant work of Shafizadeh [48,59] highlighted the central role of the porphyrin-to-metal charge-transfer (^2^D) states in the rapid formation of triplet excited states in copper porphyrins. However, for this to occur, the ^2^D state must lie at higher energy to the ^2^T_1_ state (Figure 10). The calculations presented in Figure 5 clearly indicate that for CuPh in toluene, the ^2^D state lies below the ^2^T_1_ state at all Cu–*N*(pyridine) distances, and therefore, the ^2^T_1_ state is unlikely to be populated in this system [49]. Consequently, the weak emission observed in toluene solution probably originates from the ^2^D state (Figure 6a). This explains why the emission lifetime measured in this work is relatively short (30 ns) and insensitive to the presence of triplet quenchers such as molecular oxygen.

**Figure 11:**
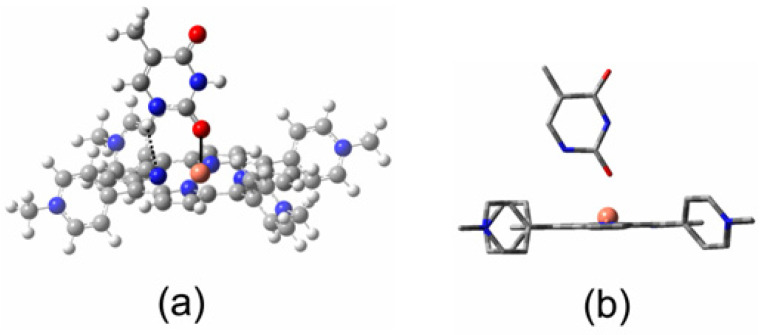
(**a**) A representation of the CuPy–thymine interaction at a Cu–O(thymine) distance of 2.145 Å in CuPy, showing the Cu–O interaction as a solid black bond and the additional endocyclic hydrogen to porphyrin nitrogen hydrogen bonding interaction as a black dashed bond, with most porphyrin atoms are rendered opaque for clarity; and (**b**) a side-on view of CuPy (wire frame, hydrogen atoms removed for clarity) showing the displacement of the copper atom from the plane of the porphyrin ring (the optimized structure was obtained using a ub3lyp/lanl2dz model chemistry).

The inset in Figure 5 clearly indicates that there is a weak interaction between the copper atom and a pyridine molecule in the ground state. Therefore, the probability that a pyridine molecule will be positioned correctly to coordinate with the copper atom at any given time will be directly dependent on the pyridine concentration. It has been noted previously that the correct orientation of the porphyrin relative to the Lewis base is essential for the coordinative expansion to occur [54]. This interaction with pyridine alters the formal d-electron configuration of the copper. This prevents the formation of the emissive state (^2^D) and therefore will manifest as a diffusive quenching despite the excited-state development occurring too fast for diffusion to play a role. Figure 6 shows the transformation of the ^2^D state into the ^2^S_0_ **_5c_** state, where the electron configuration of the copper changes to accommodate the electron donation from the pyridine ligand into the d_z_^2^ orbital of the copper. This configuration change is accompanied by a structural change, with the porphyrin with the copper atom being displaced from the porphyrin plane to form the so-called “domed” structure (Figure 11). This quasi-stable species then ejects the axial pyridine ligand in a thermal process regenerating the four-coordinate ground state.

Turning to the CuPy system, the relaxed potential energy plots in Figure 8 exhibit one significant difference compared to the plot for the CuPh system (Figure 5). The ^2^D state lies at higher energy than the ^2^T_1_ state for large Cu–donor atom distances (green plot segments). This means that in the absence of Lewis bases or in situations where the Lewis base is not correctly located to coordinate the copper atom, excitation into either the ^2^S_2_ or ^2^S_1_ state will rapidly populate to the ^2^D state, which in turn will cross to the ^2^T_1_ state (Figure 9b). At shorter Cu-to-donor atom distances and where the Lewis base is correctly orientated to coordinate to the copper atom, the ^2^D state falls below the ^2^T_1_ state, which inhibits crossing to the ^2^T_1_ state (red plot segments). The system then forms the quasi-stable five-coordinate species by relaxing into the ^2^S_0_ **_5c_** potential minimum, a process which takes some 700 fs in water (Figure 9a). This process is slower (700 fs) than the formation of the triplet state (140 fs). This means that the formation of the five-coordinate species depends not so much on the concentration of the Lewis base but on the probability that the Lewis base is correctly orientated to act as a ligand to the copper atom at the instant of excitation. This in turn explains why the triplet excited state is detected even after the five-coordinate species has relaxed the four-coordinate species. In pure water, approximately 5% of the copper porphyrin molecules are not orientated correctly for water to act as ligand to the copper atom at any given time. This results in a 5% yield of the triplet excited states even in pure water [41]. The five-coordinate species formed cannot be formally classified as an exciplex because it is formed in its lowest energy state. Water is then expelled from the adduct, regenerating the four-coordinate species in about 30 ns.

The rate of expulsion of the axial ligand from the five-coordinate species depends on the nature of the axial ligand. For example, the rate is relatively slow when thymine is the axial ligand. This is because, for thymine, additional hydrogen bonding interactions between the endocyclic hydrogen atoms and the nitrogen atoms of the porphyrin stabilize this species (Figure 11a). The copper atom is displaced from the center of the porphyrin ring because of alterations to the d-electron configuration (Figure 11b). The porphyrin ring vibrations are insensitive to the nature of the axial ligand, and it is the changes to these vibrations that are monitored in Raman studies in the 1300 to 1700 Δcm^−1^ region. This explains why simple Raman spectroscopy cannot distinguish between different five-coordinate complexes and time-resolved Raman techniques are required.

## 4. Materials and Methods

### 4.1. Transient Absorption (TA) Spectroscopy

The ultrafast (fs) transient absorption spectroscopy (UTAS) measurements reported in this work were performed using a Newport Transient Absorption Spectrometer. The spectral resolution of the system was ca. 2 nm. The laser pulses were delivered by a 1 kHz Coherent Astrella Ti:Sapphire laser system with ∼40 fs pulse duration (measured by an autocorrelator) at a repetition rate of 1 kHz and a wavelength centered at 800 nm. The second harmonic radiation, at 400 nm, provided the pump beam, and the incident laser energy on the sample was ca. 3 µJ. The white light continuum (330–900 nm) generated by means of a CaF_2_ crystal served as the probe beam. The experiments were performed at a magic angle between the polarization of the pump beam and that of the probe continuum, with a spot size in the range 150 to 200 μm. The temporal resolution of the UTAS system was estimated to be ∼120 fs and the instrument response function was approximately 90 fs.

### 4.2. Luminescence Studies

The time-resolved emission spectroscopy was carried out using an LP980 spectrometer (Edinburgh Instruments UK) coupled with a Quantel Q-smart 450 YAG laser, producing the second harmonic of the fundamental at 532 nm (8 mJ per pulse). The absorbance of the sample solution was adjusted to ca. 0.2 at 532 nm in spectroscopy-grade toluene (Merck Life Science Ltd., Arklow, Ireland), and it was monitored throughout the experiment. There was no evidence of sample photodegradation in these experiments.

### 4.3. Materials

Copper(II)-*meso*-*tetrakis*(N-methyl-pyridinium-4-yl)porphine tetrachloride and copper(II)-5,10,15,20-*meso*-*tetrakis*(phenyl)porphyrin were purchased from PorphyChem SAS. CuPh was further purified via silica gel column chromatography (3:1 pentane:dichloromethane). The oligonucleotide d(GC)_5_ was received as a gift from Professor Susan Quinn, University College Dublin, having been purchased from Kaneka Eurogentex, Seraing, Belgium. 

### 4.4. Sample Preparation

The UV-vis. and emission spectroscopy results were recorded using a Horiba Duetta Fluorescence and Absorbance spectrometer. The solutions were prepared in a 1 cm quartz fluorescence cuvette using spectroscopy grade toluene (Merck Life Science Ltd., Arklow, Ireland) or deuterium oxide 99.90% (Eurisotop, Saint-Aubin, France). The samples for the fs transient spectroscopy measurements were prepared in a demountable liquid cell (Harrick Scientific Products Inc., New York, NY, USA) with two 25 mm (2 mm thickness) CaF_2_ windows (Crystran Ltd., Poole, UK) using a 500 μm PTFE spacer. A Fisherbrand CTP100 peristatic pump was used to flow the sample during the time-resolved measurements. The CuPy samples were prepared in 18.2 MΩ MilliQ water or a 50 mM phosphate buffer (pH = 7) in D_2_O.

### 4.5. Computational Methods

All the density functional theory (DFT) calculations were performed using the Gaussian 16, Revision B.01 program suite [62]. The unrestricted hybrid density functional UB3LYP was used in the majority of calculations [63,64]. All the calculations used the double zeta quality LanL2DZ basis set, which has proved successful at modelling other inorganic systems at moderate computational cost [65,66,67]. The structures were optimized to tight convergence criteria with the exception that the pyridinium methyl groups were, when required, frozen in the latter stages of the optimization to prevent convergence problems caused by the freely rotating methyl groups. A doublet ground-state multiplicity and an overall charge of zero for the CuPh porphyrin and +4 for the CuPy porphyrin was used. The nature and energy of the higher energy states were calculated using time-dependent density functional theory (TD-DFT) methods [68,69,70,71,72,73]. The calculations were modelled for the presence of toluene in the case of CuPh and water in the case of CuPy using the polarizable continuum model [74,75]. GaussView (revision 6.0.16) was used to visualize the results of the quantum calculations [76].

## 5. Conclusions

**Figure 12:**
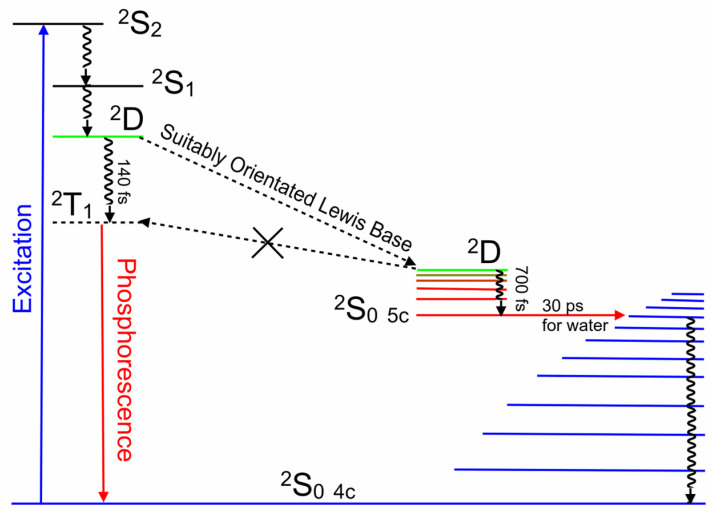
An energy-level representation of the photophysical processes following 400 nm excitation of CuPy, describing the population of the ^2^T_1_ state in the absence of a suitably orientated Lewis base while the five-coordinate species provides a deactivation route to the four-coordinate species.

These results highlight the important role of a charge-transfer state in directing the photophysics of copper porphyrins. Both the solvent and the nature of the substituents in the porphyrin *meso*-positions can affect the energy of this state and therefore change the direction of the excited-state development. It is incorrect to assume that a porphyrin with *meso*-phenyl substituents will behave in a similar way to porphyrins containing *meso*-pyridinium groups. Solvents can affect the photophysics of these porphyrins by altering the energy of the ^2^D ligand-to-metal charge-transfer state through changes to the medium polarity, and also via their Lewis base character. For the CuPy system, the presence of a Lewis base donor atom close to the copper atom can divert the system away from crossing to the triplet surface by opening a route to coordinative expansion. This forms a ground-state five-coordinate species. Both triplet formation and coordinative expansion occur on the ultrafast timescale of 140 fs and 700 fs, respectively, too fast for bimolecular diffusion to play a significant role. Consequently, copper porphyrins are excellent probes in the immediate environment of the copper atom at the instant of excitation. Figure 12 summarizes the excited-state development of these copper porphyrin systems.

## Data Availability

Not applicable.

## Supplementary Materials

The following supporting information can be downloaded at https://www.mdpi.com/article/10.3390/molecules28176310/s1, Table S1. Optimized atomic coordinates of the CuPh in toluene; Table S2. Optimized atomic coordinates of the CuPy in water; Table S3. Valence orbitals with fragment contributions (%) for CuPh; Table S4. Summary of the calculated electronic transitions for CuPh; Table S5. Orbital fragment contributions (%) for CuPy; Table S6. Summary of the calculated electronic transitions for CuPy; Table S7. Optimized atomic coordinates for CuPy–thymine; Table S8. Optimized bond lengths and angles for CuPy–thymine; Figure S1. The adiabatic plots for the lowest 20 excited states along the Cu–N(pyridine) reaction coordinate; Figure S2. The adiabatic plots for the lowest 20 excited states along the Cu–O(water) reaction coordinate; Figure S4. The electron density difference maps the Q-band excited states; Figure S5. The electron density difference maps for the B-band excited states.

## Sample Availability

Samples of the compounds are not available from the authors.
