# Supplementary material for: Excited-State Dynamics Leading Either to Triplet Formation or Coordinative Expansion following Photolysis of Cu(II)-Porphyrins: A DFT, TD-DFT, Luminescence and Femtosecond Time-Resolved Absorbance Study
Source: Molecules. 2023 Aug 29;28(17):6310. doi: 10.3390/molecules28176310 (PMC10488807; doi:10.3390/molecules28176310)
Supplement: Supplementary file 1 [file molecules-28-06310-s001.zip › molecules-2539127-supplementary.pdf]

Supporting Information

Excited-state Dynamics Leading either to Triplet Formation or Coordinative Expansion following Photolysis of Cu(II)-porphyrins: a DFT, TD-DFT, Luminescence and Femtosecond Time-Resolved Absorbance Study

Ross J. McGarry, Lazaros Varvarezos, Mary T. Pryce, and Conor Long

Contents

		PAGE
**Table S1.**	Optimized atomic coordinates of the CuPh in toluene	2
**Table S2.**	Optimized atomic coordinates of the CuPy in water	4
**Figure S1.**	The adiabatic plots for the lowest 20 excited states along the Cu-N(pyridine) reaction coordinate	6
**Figure S2**.	The adiabatic plots for the lowest 20 excited states along the Cu-O(water) reaction coordinate	6
**Table S3**.	Valence orbitals with fragment contributions (%) for CuPh	7
**Table S4.**	Summary of calculated electronic transitions for CuPh	8
**Figure S3.**	The electron density difference map for the LMCT state in CuPh	9
**Figure S4.**	The electron density difference maps the Q band excited-states	10
**Figure S5.**	The electron density difference maps for the B band excited states	11
**Table S5**.	Orbital Fragment contributions (%) for CuPy	12
**Table S6**.	Summary of calculated electronic transitions for CuPy (f = Osc Str.)	13
**Table S7.**	Optimized atomic coordinates for CuPy-thymine	14
**Table S8.**	Optimized Bond-Lengths and Angles for CuPy-thymine	17

Table S1. Optimized (B3LYP/LanL2DZ) atomic coordinates of the CuPh in toluene

	x	y	z
N	-1.56218	1.316803	-0.00505
C	-2.91988	0.996966	-0.05982
C	-1.47672	2.709022	-0.06117
C	-3.69817	2.2194	-0.19165
C	-2.81327	3.269213	-0.19254
H	-4.7727	2.261944	-0.28711
H	-3.03708	4.320967	-0.28884
N	1.316172	1.561201	0.005264
C	2.708547	1.47599	0.061372
C	0.996338	2.919065	0.060069
C	3.26855	2.81257	0.192894
C	2.218707	3.69738	0.19192
H	4.320274	3.036461	0.289264
H	2.261163	4.771916	0.287342
N	-1.31617	-1.56132	0.005194
C	-2.70854	-1.476	0.061294
C	-0.99639	-2.91915	0.060034
C	-3.26858	-2.81259	0.192769
C	-2.21877	-3.69743	0.191931
H	-4.32031	-3.03647	0.289116
H	-2.26125	-4.77196	0.287437
N	1.562097	-1.31659	-0.00508
C	1.476631	-2.70884	-0.06121
C	2.91982	-0.99685	-0.05993
C	2.813177	-3.26906	-0.1926
C	3.698104	-2.21928	-0.19172
H	3.036959	-4.32082	-0.28885
H	4.772645	-2.26182	-0.28712
C	3.477479	0.296208	0.001143
C	4.974779	0.4238	0.002337
C	5.649461	1.019855	-1.08811
C	5.73844	-0.05007	1.094019
C	7.052188	1.135597	-1.08982
H	5.075402	1.379496	-1.93857
C	7.14057	0.072483	1.098342
H	5.231704	-0.50174	1.943356
H	7.554364	1.589447	-1.94082
H	7.71053	-0.29031	1.950337
C	0.296525	-3.47724	-0.00107
C	0.423692	-4.97458	-0.00234
C	-0.0504	-5.73809	-1.09404
C	1.01991	-5.64941	1.087913

C	0.071986	-7.14023	-1.0985
H	-0.50213	-5.23121	-1.94326
C	1.13551	-7.05215	1.089476
H	1.37986	-5.07545	1.938311
H	-0.291	-7.71007	-1.95049
H	1.5895	-7.55445	1.94033
C	-3.47745	-0.29617	0.001168
C	-4.97477	-0.42383	0.002331
C	-5.64942	-1.01984	-1.08816
C	-5.73846	0.049833	1.094086
C	-7.05214	-1.13568	-1.08988
H	-5.07535	-1.37936	-1.93866
C	-7.14058	-0.07283	1.098413
H	-5.23175	0.50141	1.943486
H	-7.55429	-1.58947	-1.94092
H	-7.71055	0.289819	1.950459
C	-0.29653	3.47728	-0.0011
C	-0.42363	4.974643	-0.00236
C	0.05038	5.738121	-1.09411
C	-1.01974	5.649477	1.087951
C	-0.07197	7.140262	-1.09857
H	0.502049	5.231233	-1.94336
C	-1.13527	7.052227	1.089535
H	-1.37958	5.07553	1.938406
H	0.290943	7.7101	-1.95059
H	-1.58916	7.554536	1.940438
Cu	-4.8E-05	0.000025	0.000023
C	0.663822	-7.80331	-0.00525
H	0.75578	-8.88675	-0.00634
C	7.803492	0.664225	0.004938
H	8.886925	0.756294	0.005914
C	-0.6637	7.803359	-0.00526
H	-0.75564	8.886804	-0.00636
C	-7.80347	-0.66448	0.00494
H	-8.88689	-0.75662	0.005916

Table S2. Optimized (B3LYP/LanL2DZ) atomic coordinates of the CuPy in water

	x	y	z
N	-0.74748	-1.7562	0.083094
C	-0.08144	-2.98168	0.081145
C	-2.10954	-2.05816	0.059577
C	-1.04843	-4.06917	0.040815
C	-2.29523	-3.50148	0.035466
H	-0.81481	-5.12246	0.011721
H	-3.24356	-4.01684	0.022511
N	-1.75622	0.936099	0.104335
C	-2.05974	2.297443	0.101645
C	-2.9812	0.268474	0.129835
C	-3.50338	2.481546	0.138756
C	-4.06986	1.234253	0.149035
H	-4.01936	3.429209	0.163166
H	-5.12318	0.998888	0.161693
N	1.94512	-0.74668	0.0704
C	2.246376	-2.10875	0.103567
C	3.170058	-0.08008	0.108147
C	3.687848	-2.29329	0.188338
C	4.255463	-1.04669	0.191621
H	4.200553	-3.24028	0.261406
H	5.306165	-0.81244	0.269077
N	0.936169	1.945248	0.12352
C	2.297668	2.24784	0.092177
C	0.268256	3.169869	0.086826
C	2.480881	3.689768	0.00976
C	1.23384	4.256289	0.006429
H	3.427233	4.203626	-0.06267
H	0.998532	5.307004	-0.06776
C	-1.1266	3.350209	0.087728
C	-1.65041	4.752483	0.070157
C	-2.38191	5.248451	-1.03451
C	-1.42831	5.630274	1.154484
C	-2.85236	6.55851	-1.03233
H	-2.57237	4.629639	-1.90299
C	-1.92527	6.932606	1.118133
H	-0.88629	5.306826	2.034796
H	-3.40545	6.975815	-1.86388
H	-1.78166	7.62551	1.936444
C	3.351297	1.315312	0.102084
C	4.752434	1.840467	0.102967
C	5.632294	1.583248	-0.97513
C	5.2464	2.611575	1.179037
C	6.930348	2.084945	-0.95628
H	5.309296	1.011273	-1.83647

C	6.556868	3.087624	1.160212
H	4.628181	2.831126	2.041032
H	7.627912	1.916286	-1.76666
H	6.968654	3.670981	1.973033
C	1.313765	-3.16156	0.096358
C	1.840716	-4.56256	0.111921
C	2.587453	-5.0752	-0.97199
C	1.611359	-5.42031	1.213447
C	3.066702	-6.38416	-0.93848
H	2.786441	-4.47264	-1.84994
C	2.114879	-6.71806	1.208134
H	1.05909	-5.08076	2.081272
H	3.632653	-6.81053	-1.75601
H	1.9667	-7.39996	2.035669
C	-3.16082	-1.12548	0.097039
C	-4.564	-1.65056	0.101549
C	-5.1101	-2.28918	-1.03592
C	-5.38829	-1.52435	1.241245
C	-6.41839	-2.76452	-1.01115
H	-4.53146	-2.40619	-1.94424
C	-6.69149	-2.0213	1.224349
H	-5.02276	-1.05635	2.147298
H	-6.87334	-3.25069	-1.86442
H	-7.34552	-1.94846	2.083005
N	-2.62426	7.381985	0.03507
N	-7.19089	-2.6296	0.108923
N	2.829881	-7.18595	0.140728
N	7.379031	2.82477	0.102592
C	-8.59171	-3.15352	0.089783
H	-9.05021	-2.99028	1.064967
H	-9.1621	-2.62391	-0.67689
H	-8.57229	-4.22301	-0.13009
C	-3.16206	8.777315	0.002827
H	-2.81418	9.318142	0.882793
H	-2.80377	9.279431	-0.89831
H	-4.25399	8.740717	0.001681
C	8.783955	3.337013	0.085923
H	8.929997	3.953012	-0.80397
H	8.958863	3.937952	0.978071
H	9.474552	2.490693	0.072729
C	3.344231	-8.58992	0.16889
H	3.919556	-8.78157	-0.73653
H	3.985525	-8.71861	1.043333
H	2.499196	-9.28053	0.21951
Cu	0.094387	0.094596	0.095457

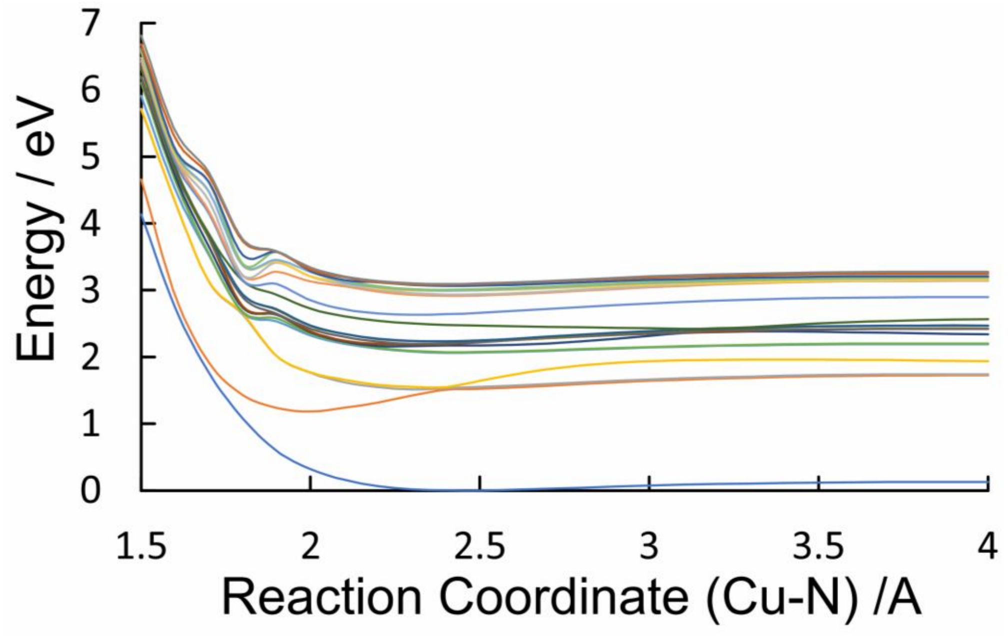

Figure S1. The adiabatic plots for the lowest 20 excited states along the Cu-N(pyridine) reaction coordinate.

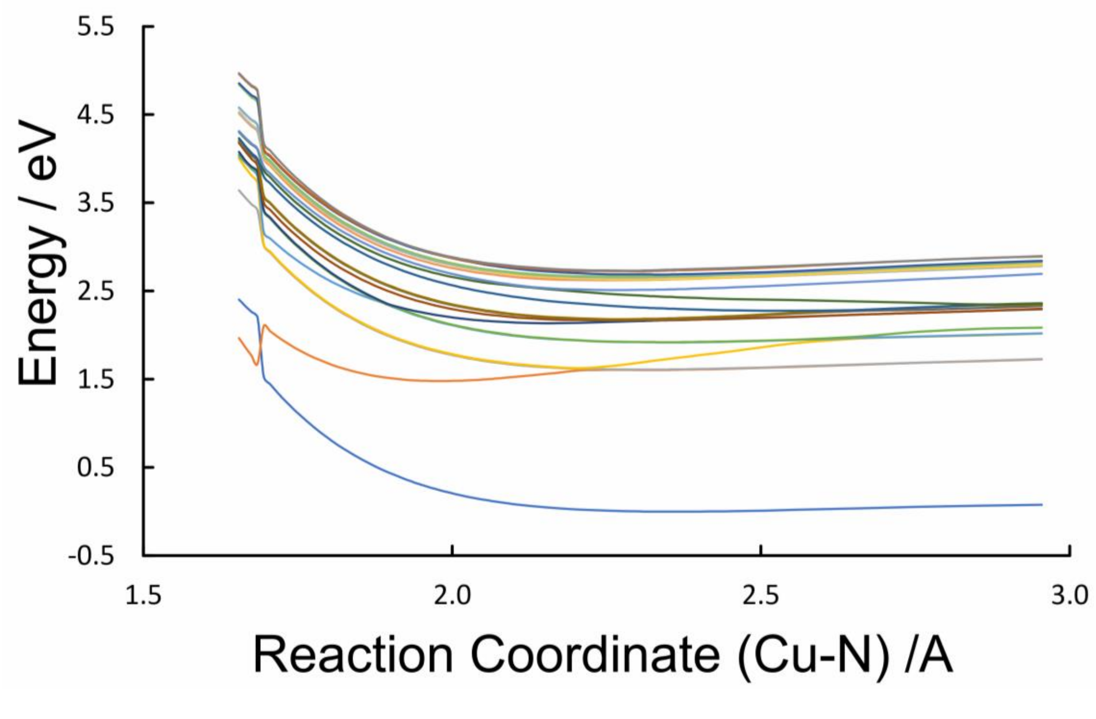

Figure S2. The adiabatic plots for the lowest 20 excited states along the Cu-O(water) reaction coordinate

Table S3. Valence orbitals with fragment contributions (%) for CuPh

Alpha MO:	161	162	163	164	165	166	167	168	169	170
	HOMO-9	HOMO-8	HOMO-7	HOMO-6	HOMO-5	HOMO-4	HOMO-3	HOMO-2	HOMO-1	HOMO
Energy(eV):	-7.11	-6.95	-6.93	-6.93	-6.79	-6.78	-6.78	-6.50	-5.57	-5.31
Symmetry:	A	A	A	A	A	A	A	A	A	A
Copper :	0.27	0.00	0.24	0.24	0.01	2.69	2.70	21.10	0.00	1.63
Porphyrin :	5.49	7.81	21.00	20.99	86.48	87.41	87.41	78.58	97.77	86.21
Phenyl :	94.24	92.19	78.76	78.77	13.51	9.90	9.89	0.32	2.23	12.16

Alpha MO:	171	172	173	174	175	176	177	178	179	180
	LUMO	LUMO+1	LUMO+2	LUMO+3	LUMO+4	LUMO+5	LUMO+6	LUMO+7	LUMO+8	LUMO+9
Energy(eV):	-2.53	-2.53	-1.01	-0.50	-0.48	-0.48	-0.43	-0.41	-0.40	-0.39
Symmetry:	A	A	A	A	A	A	A	A	A	A
Copper :	0.32	0.32	0.00	0.39	0.66	0.66	0.00	0.21	0.00	0.47
Porphyrin :	94.15	94.15	84.66	4.67	4.56	4.56	2.90	0.74	16.92	3.40
Phenyl :	5.52	5.53	15.34	94.93	94.78	94.78	97.10	99.05	83.08	96.12

Beta MO:	161	162	163	164	165	166	167	168	169	170
	HOMO-8	HOMO-7	HOMO-6	HOMO-5	HOMO-4	HOMO-3	HOMO-2	HOMO-1	HOMO	LUMO
Energy(eV):	-7.10	-6.95	-6.93	-6.93	-6.74	-6.70	-6.70	-5.59	-5.28	-2.57
Symmetry:	A	A	A	A	A	A	A	A	A	A
Copper :	0.27	0.00	0.21	0.21	0.01	4.14	4.16	0.00	1.94	59.90
Porphyrin :	3.56	7.81	18.42	18.42	89.69	90.46	90.46	97.76	86.06	40.08
Phenyl :	96.17	92.19	81.37	81.37	10.30	5.40	5.39	2.24	12.00	0.02

Beta MO:	171	172	173	174	175	176	177	178	179	180
	LUMO+1	LUMO+2	LUMO+3	LUMO+4	LUMO+5	LUMO+6	LUMO+7	LUMO+8	LUMO+9	LUMO+10
Energy(eV):	-2.51	-2.51	-1.01	-0.50	-0.48	-0.48	-0.43	-0.41	-0.40	-0.39
Symmetry:	A	A	A	A	A	A	A	A	A	A
Copper :	0.41	0.41	0.00	0.44	0.68	0.67	0.00	0.19	0.00	0.46
Porphyrin :	93.92	93.92	84.63	4.66	4.53	4.54	2.91	0.73	16.95	3.48
Phenyl :	5.67	5.67	15.37	94.90	94.79	94.79	97.09	99.08	83.05	96.06

Table S4. Summary of calculated electronic transitions for CuPh (f = oscillator strength)

#	nm	1000 cm-1	eV	f	Orbital Assignment (excitations with contrib. greater than 10.0%)
1	775.1	12.90	1.599	0.0000	169->172B(40.6%) 170->172(40.4%)
2	775.1	12.90	1.600	0.0000	169->171B(40.6%) 170->171(40.4%)
3	709.9	14.09	1.747	0.0000	169->170B(98.5%)
4	598.7	16.70	2.071	0.0000	169->171(49.5%) 168->171B(41.6%)
5	598.6	16.70	2.071	0.0000	169->172(49.5%) 168->172B(41.6%)
6	575.3	17.38	2.155	0.0000	168->170B(99.9%)
7	543.0	18.42	2.283	0.0493	170->171(33.3%) 169->171B(31.3%) 168->172B(19.5%) 169->172(13.6%)
8	542.9	18.42	2.284	0.0492	170->172(33.3%) 169->172B(31.3%) 168->171B(19.5%) 169->171(13.6%)
9	531.8	18.81	2.332	0.0003	167->170B(47.4%) 146->170B(45.3%)
10	531.5	18.81	2.333	0.0003	166->170B(47.4%) 147->170B(45.4%)
11	504.1	19.84	2.459	0.0000	151->170B(93.5%)
12	447.6	22.34	2.770	0.0000	137->170B(71.5%) 148->170B(21.1%)
13	411.2	24.32	3.015	0.0000	166->171B(18.4%) 167->172B(18.2%) 166->171(13.2%) 167->172(13.1%)
14	410.2	24.38	3.023	1.1047	168->171B(26.2%) 169->171(24.6%) 168->172(19.5%) 169->172B(12.4%) 170->172(12.1%)
15	410.2	24.38	3.023	1.1023	168->172B(26.2%) 169->172(24.6%) 168->171(19.6%) 169->171B(12.4%) 170->171(12.0%)
16	401.5	24.90	3.088	0.0000	166->171B(22.4%) 167->172B(22.4%) 166->171(12.5%) 167->172(12.5%)
17	400.9	24.94	3.093	0.3242	168->171(76.1%)
18	400.9	24.94	3.093	0.3224	168->172(76.3%)
19	396.8	25.20	3.125	0.0000	167->171B(23.2%) 166->172B(23.1%) 167->171(15.3%) 166->172(15.2%)
20	393.3	25.42	3.152	0.0001	169->173B(31.8%) 170->173(28.9%)
21	393.3	25.43	3.153	0.0271	165->171B(37.0%) 165->171(33.0%)
22	393.2	25.43	3.154	0.0266	165->172B(37.1%) 165->172(33.1%)
23	385.6	25.93	3.215	0.0002	165->170B(88.6%)
24	380.8	26.26	3.256	0.0000	166->172B(23.0%) 167->171B(23.0%) 166->172(16.9%) 167->171(16.8%)
25	353.0	28.33	3.512	0.0000	166->171B(21.4%) 167->172B(21.3%) 166->171(20.2%) 167->172(20.1%)
26	351.6	28.44	3.527	0.0000	
27	350.1	28.57	3.542	0.0000	169->173(28.5%) 168->173B(27.6%)
28	348.4	28.70	3.558	0.0000	167->172(24.8%) 166->171(24.7%) 167->172B(21.7%) 166->171B(21.6%)
29	347.2	28.80	3.571	0.0001	
30	347.2	28.80	3.571	0.0001	
31	343.7	29.09	3.607	0.0000	
32	342.3	29.21	3.622	0.0022	156->172(14.1%) 159->172B(13.1%) 156->172B(10.8%) 157->172(10.2%)
33	342.3	29.22	3.622	0.0022	156->171(14.5%) 159->171B(13.5%) 156->171B(11.1%) 157->171(10.7%)
34	340.6	29.36	3.640	0.0012	166->170B(39.7%) 147->170B(30.9%)
35	340.5	29.37	3.641	0.0013	167->170B(40.7%) 146->170B(31.7%)
36	340.1	29.40	3.645	0.0036	165->171(52.1%) 165->171B(41.4%)
37	340.1	29.40	3.645	0.0034	165->172(52.3%) 165->172B(41.3%)
38	337.2	29.66	3.677	0.0000	159->170B(75.1%) 156->170B(22.5%)
39	337.0	29.67	3.679	0.0000	167->171(25.6%) 166->172(25.4%) 167->171B(20.6%) 166->172B(20.4%)
40	329.8	30.33	3.760	0.0000	166->172B(22.0%) 167->171B(21.9%) 166->172(19.4%) 167->171(19.2%)

Red background 2D state; Yellow Q Bands; Green B Bands

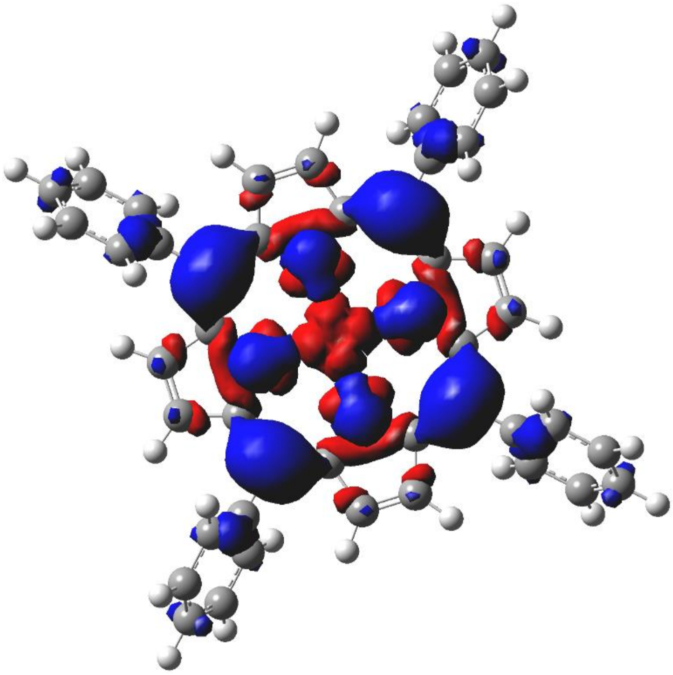

Figure S3. The electron density difference map for the LMCT state in CuPh (ES3) which is not optically populated showing considerable *meso*-carbon-to-d_{x2-y2} character; Blue volumes are the regions where the electron density is less in the excited-state compared to the ground-state and the red regions are the regions where the electron density is greater in the excited-state compared to the ground-state.

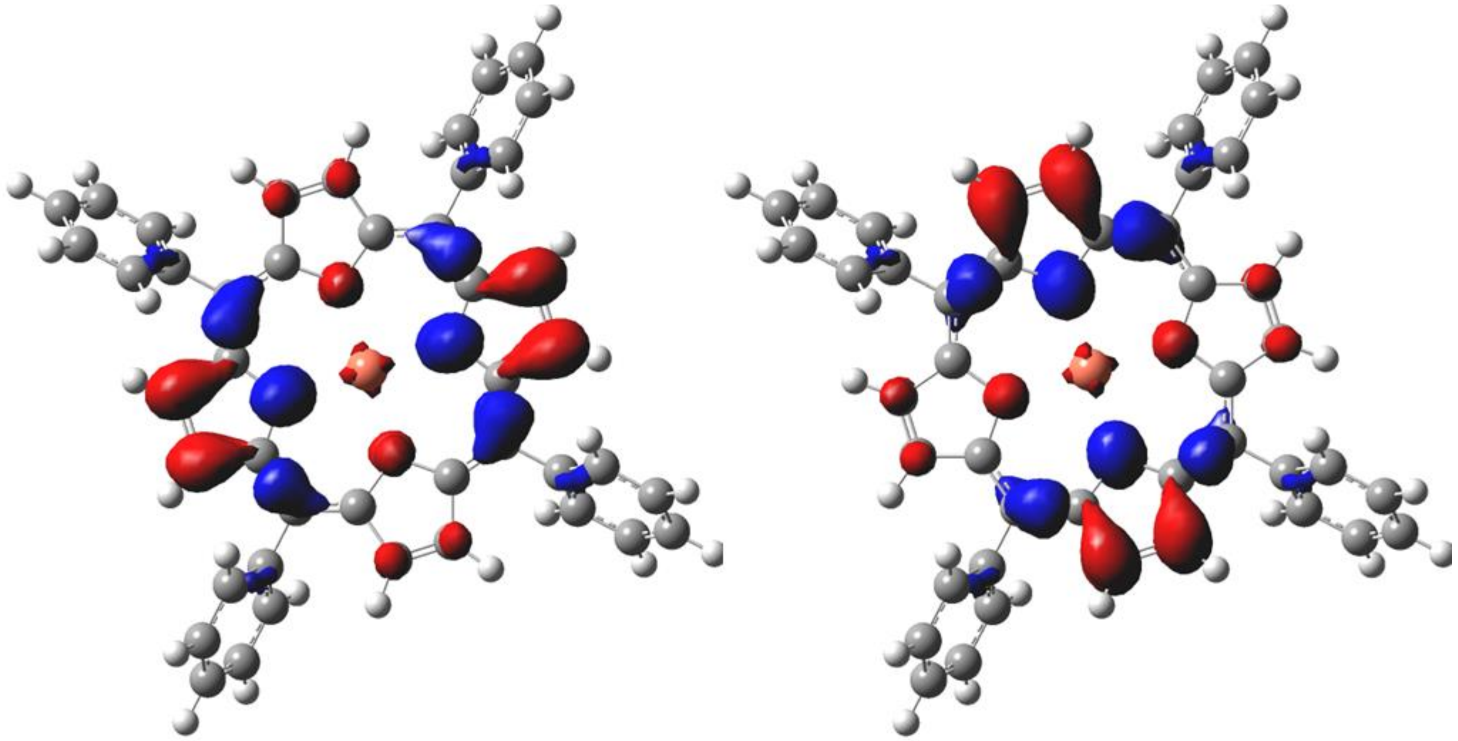

ES7 ES8

Figure S4. The electron density difference maps for the degenerate pair comprising the Q band excited-states, volume colours are as in Figure S 3

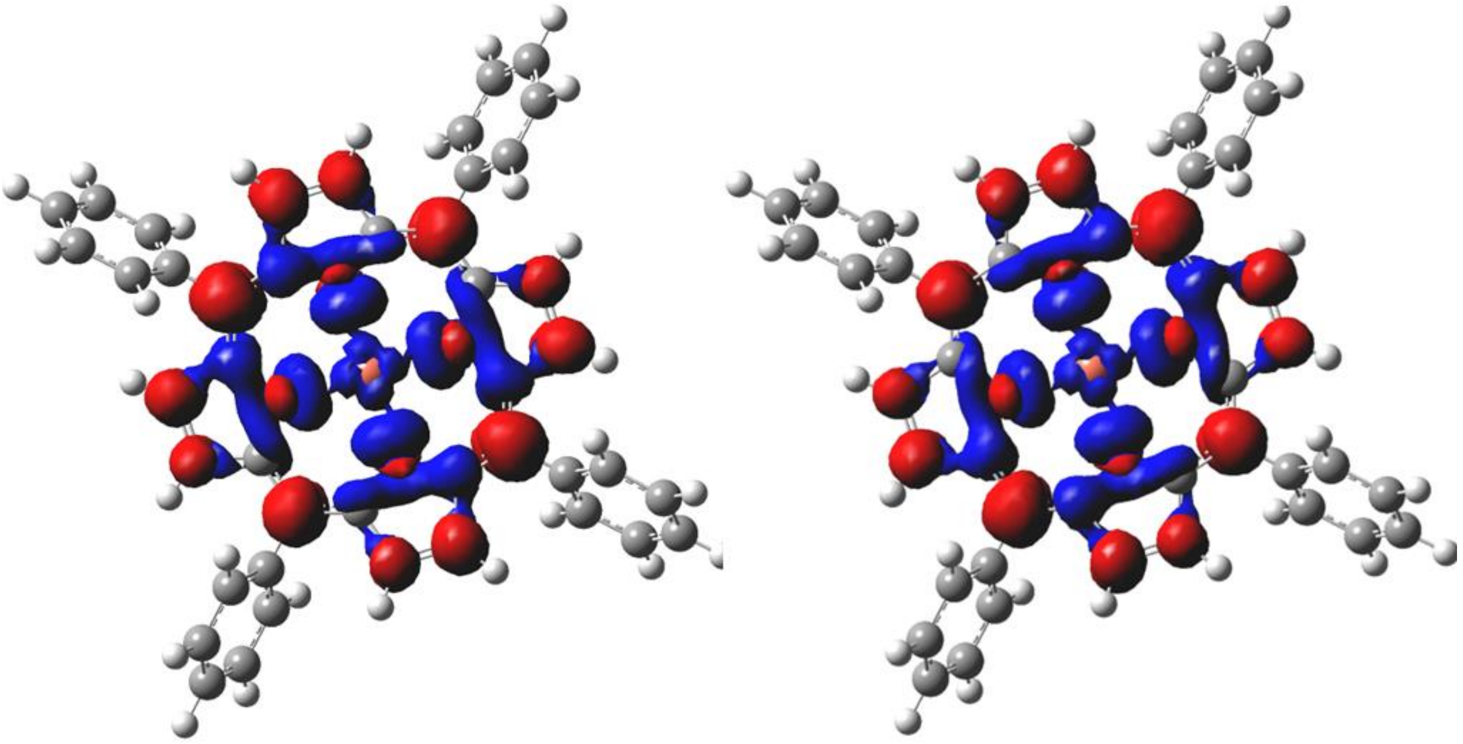

ES14 ES15

Figure S5. The electron density difference maps for the B band excited states, volume colours are as in Figure S3.

Table S5. Orbital Fragment contributions (%) for CuPy

Summary of calculated valence orbitals with fragment contributions for CuPy:

Alpha MO:	171	172	173	174	175	176	177	178	179	180
	HOMO-15	HOMO-14	HOMO-13	HOMO-12	HOMO-11	HOMO-10	HOMO-9	HOMO-8	HOMO-7	HOMO-6
Energy(eV):	-9.45	-9.36	-9.13	-8.88	-8.88	-8.87	-8.86	-8.43	-8.43	-7.90
Symmetry:	A	A	A	A	A	A	A	A	A	A
Cu 1:	0.69	0.13	0.03	0.02	0.03	0.00	0.01	0.03	0.03	1.96
Porphyrin 2:	27.60	15.82	30.70	3.16	3.79	0.95	1.60	82.58	82.48	97.53
Pyridinium3:	71.70	84.04	69.28	96.83	96.18	99.04	98.39	17.39	17.49	0.51

Alpha MO:	181	182	183	184	185	186	187	188	189	190
	HOMO-5	HOMO-4	HOMO-3	HOMO-2	HOMO-1	HOMO	LUMO	LUMO+1	LUMO+2	LUMO+3
Energy(eV):	-7.53	-7.51	-7.51	-7.25	-6.32	-6.29	-3.49	-3.48	-3.02	-2.89
Symmetry:	A	A	A	A	A	A	A	A	A	A
Cu 1:	0.01	3.20	3.20	21.81	0.00	1.42	0.22	0.24	-0.01	0.17
Porphyrin 2:	99.17	95.80	95.82	78.18	98.66	89.01	71.13	71.75	6.62	4.86
Pyridinium3:	0.82	0.99	0.98	0.02	1.34	9.57	28.65	28.02	93.39	94.96

Alpha MO:	191	192	193	194	195	196	197	198	199	200
	LUMO+4	LUMO+5	LUMO+6	LUMO+7	LUMO+8	LUMO+9	LUMO+10	LUMO+11	LUMO+12	LUMO+13
Energy(eV):	-2.76	-2.76	-1.94	-1.93	-1.91	-1.91	-1.62	0.08	0.31	0.64
Symmetry:	A	A	A	A	A	A	A	A	A	A
Cu 1:	0.11	0.09	0.32	0.03	0.36	0.09	0.00	0.43	94.39	90.84
Porphyrin 2:	26.34	26.90	1.44	1.08	2.96	2.60	93.80	98.77	4.36	5.65
Pyridinium3:	73.55	73.01	98.24	98.89	96.67	97.31	6.20	0.80	1.25	3.51

Beta MO:	171	172	173	174	175	176	177	178	179	180
	HOMO-14	HOMO-13	HOMO-12	HOMO-11	HOMO-10	HOMO-9	HOMO-8	HOMO-7	HOMO-6	HOMO-5
Energy(eV):	-9.32	-9.31	-9.13	-8.88	-8.88	-8.87	-8.86	-8.44	-8.43	-7.86
Symmetry:	A	A	A	A	A	A	A	A	A	A
Cu 1:	1.28	86.90	0.11	0.03	0.04	0.01	0.02	0.03	0.04	1.98
Porphyrin 2:	49.37	13.06	31.60	3.21	3.89	0.96	1.60	82.60	82.48	97.52
Pyridinium3:	49.34	0.05	68.30	96.76	96.06	99.03	98.39	17.36	17.48	0.51

Beta MO:	181	182	183	184	185	186	187	188	189	190
	HOMO-4	HOMO-3	HOMO-2	HOMO-1	HOMO	LUMO	LUMO+1	LUMO+2	LUMO+3	LUMO+4
Energy(eV):	-7.48	-7.43	-7.43	-6.34	-6.26	-3.48	-3.48	-3.24	-3.02	-2.89
Symmetry:	A	A	A	A	A	A	A	A	A	A
Cu 1:	0.01	4.66	4.66	0.00	1.62	0.28	1.19	59.56	0.28	0.20
Porphyrin 2:	99.23	94.42	94.43	98.66	88.75	69.74	69.91	39.54	6.77	4.93
Pyridinium3:	0.77	0.92	0.91	1.34	9.63	29.98	28.91	0.90	92.95	94.87

Beta MO:	191	192	193	194	195	196	197	198	199	200
	LUMO+5	LUMO+6	LUMO+7	LUMO+8	LUMO+9	LUMO+10	LUMO+11	LUMO+12	LUMO+13	LUMO+14
Energy(eV):	-2.75	-2.75	-1.94	-1.93	-1.91	-1.91	-1.62	0.13	0.30	0.64
Symmetry:	A	A	A	A	A	A	A	A	A	A
Cu 1:	0.14	0.12	0.32	0.03	0.36	0.09	0.00	0.49	94.03	92.49
Porphyrin 2:	27.51	28.06	1.46	1.06	3.05	2.68	93.78	98.67	4.70	3.91
Pyridinium3:	72.35	71.82	98.22	98.91	96.59	97.23	6.22	0.84	1.27	3.60

Table S6. Summary of calculated electronic transitions for CuPy (f = Osc Str.)

#	nm	1000 cm-1	eV	f	Assignment (excitations with contrib. greater than 10.0%)
1	743.3	13.45	1.668	0.0001	185->186B(34.4%) 186->187(33.6%) 185->188(11.6%) 184->187B(11.3%)
2	742.7	13.46	1.669	0.0001	185->187B(33.8%) 186->188(32.9%) 185->187(12.2%) 184->186B(11.9%)
3	637.1	15.70	1.946	0.0000	185->187(37.9%) 184->186B(32.1%) 185->187B(15.0%) 186->188(10.9%)
4	636.0	15.72	1.950	0.0000	185->188(38.2%) 184->187B(33.4%) 185->186B(13.6%) 186->187(10.9%)
5	608.5	16.43	2.037	0.0006	185->188B(95.3%)
6	559.1	17.89	2.218	0.0057	184->188B(63.5%) 184->187B(11.7%)
7	553.6	18.06	2.240	0.0234	186->188(28.2%) 185->187B(24.4%) 184->186B(24.3%) 185->187(19.7%)
8	552.1	18.11	2.246	0.0249	184->188B(35.3%) 186->187(21.6%) 185->186B(18.5%) 184->187B(12.2%) 185->188(11.1%)
9	531.6	18.81	2.332	0.0000	164->188B(36.3%) 183->188B(31.7%) 182->188B(15.9%) 163->188B(10.6%)
10	531.5	18.81	2.333	0.0000	163->188B(35.9%) 182->188B(31.7%) 183->188B(15.9%) 164->188B(10.6%)
11	510.7	19.58	2.428	0.0000	172->188B(92.0%)
12	468.5	21.35	2.647	0.0000	185->189B(60.0%) 186->189(34.3%)
13	455.7	21.94	2.721	0.6826	185->187(19.8%) 184->186B(19.6%) 185->187B(17.1%) 186->188(14.5%) 185->191B(12.2%)
14	455.5	21.95	2.722	0.6995	185->188(20.8%) 184->187B(20.2%) 185->186B(17.0%) 186->187(14.2%) 185->192B(12.0%)
15	450.7	22.19	2.751	0.0001	185->189(55.7%) 184->189B(40.5%)
16	446.2	22.41	2.779	0.0111	186->189(60.5%) 185->189B(36.1%)
17	445.1	22.47	2.786	0.0000	162->188B(72.2%) 158->188B(24.0%)
18	444.0	22.52	2.792	0.0024	185->190B(56.5%) 186->190(32.8%)
19	437.1	22.88	2.837	0.0012	184->189B(56.5%) 185->189(41.7%)
20	436.4	22.92	2.841	0.0068	186->191(38.5%) 185->191B(38.1%)
21	436.2	22.93	2.843	0.0078	186->192(41.3%) 185->192B(40.0%)
22	430.2	23.25	2.882	0.0000	183->186B(18.7%) 182->187B(17.7%) 183->187(14.1%) 182->188(13.7%)
23	426.7	23.44	2.906	0.0001	185->190(45.7%) 184->190B(20.2%)
24	424.9	23.54	2.918	0.0004	186->190(62.4%) 185->190B(35.9%)
25	423.5	23.61	2.928	0.0002	185->190(27.3%) 182->186B(14.4%) 183->187B(13.7%)
26	422.0	23.69	2.938	0.0022	184->187(89.2%)
27	421.2	23.74	2.944	0.0014	184->188(92.9%)
28	420.7	23.77	2.947	0.0015	184->190B(70.4%) 185->190(23.8%)
29	418.0	23.92	2.966	0.0027	185->191(46.9%) 184->191B(26.5%)
30	417.7	23.94	2.968	0.0022	185->192(44.8%) 184->192B(24.2%)
31	411.2	24.32	3.015	0.0008	181->187B(18.6%) 181->188(14.1%) 184->191B(13.2%) 181->186B(10.4%)
32	411.2	24.32	3.015	0.0005	181->186B(17.2%) 184->192B(13.9%) 181->187(13.7%) 181->187B(10.8%)
33	410.3	24.37	3.022	0.0236	186->191(29.3%) 184->192B(27.1%) 185->192(19.6%) 185->191B(15.2%)
34	410.2	24.38	3.023	0.0293	186->192(27.9%) 184->191B(26.2%) 185->191(22.0%) 185->192B(12.7%)
35	408.8	24.46	3.033	0.0000	182->187B(21.2%) 183->186B(20.4%) 182->188(15.6%) 183->187(14.6%)
36	394.7	25.33	3.141	0.0000	183->187B(21.2%) 182->186B(21.0%) 183->188(14.0%) 182->187(13.5%)
37	379.7	26.34	3.265	0.0030	181->188B(94.2%)
38	374.8	26.68	3.308	0.7057	184->192B(19.1%) 185->192(16.3%) 186->191(14.4%) 185->191B(13.7%)
39	374.8	26.68	3.308	0.6922	184->191B(17.5%) 186->192(15.5%) 185->191(15.0%) 185->192B(14.6%)
40	374.4	26.71	3.312	0.0047	182->187(24.7%) 183->188(21.7%) 182->186B(19.7%) 183->187B(17.5%)
41	370.8	26.97	3.344	0.0005	183->187(21.8%) 182->188(19.3%) 183->186B(18.3%) 182->187B(16.1%)
42	361.7	27.65	3.428	0.0082	181->186B(48.3%) 181->187(43.0%)
43	360.8	27.71	3.436	0.0077	181->187B(42.6%) 181->188(37.7%)
44	360.1	27.77	3.443	0.0000	185->197B(34.7%) 186->197(31.9%)
45	358.4	27.90	3.460	0.0001	183->188(21.7%) 182->187(18.2%) 183->187B(15.0%) 182->186B(12.3%)
46	358.0	27.93	3.463	0.0000	180->186B(29.0%) 180->187(22.3%) 181->188(12.0%)
47	357.9	27.94	3.464	0.0000	180->187B(30.5%) 180->188(24.3%)
48	353.0	28.33	3.512	0.0000	182->188(24.5%) 183->187(21.7%) 182->187B(20.8%) 183->186B(18.8%)
49	336.6	29.71	3.683	0.0000	185->197(35.4%) 184->197B(34.5%)
50	333.9	29.95	3.713	0.0001	183->188B(27.9%) 163->188B(24.5%) 182->188B(21.2%) 164->188B(20.2%)

Red ^2D state Yellow Q Bands, Green B Bands, Grey $^2T_1$ states, Purple $^4T_1$ states

Table S7. The Optimized atomic coordinates for CuTMPyP4-(κ-*O*-thymine)

Atom No.	Symbol	X	Y	Z
1	N	1.858019	0.010428	-0.742
2	C	2.683601	-1.10241	-0.91408
3	C	2.665498	1.136657	-0.89998
4	C	4.053825	-0.658	-1.15024
5	C	4.041008	0.716363	-1.15113
6	H	4.913636	-1.29271	-1.30374
7	H	4.88379	1.367565	-1.32949
8	N	-0.23902	2.069695	-0.70693
9	C	-1.36007	2.88594	-0.63604
10	C	0.86541	2.908439	-0.80857
11	C	-0.94736	4.289318	-0.70032
12	C	0.420732	4.303401	-0.79901
13	H	-1.60087	5.148802	-0.68929
14	H	1.053106	5.176873	-0.85086
15	N	-0.204	-2.08359	-0.64446
16	C	0.914085	-2.90047	-0.77244
17	C	-1.31381	-2.9172	-0.63244
18	C	0.489863	-4.29842	-0.85778
19	C	-0.87966	-4.30912	-0.77217
20	H	1.135829	-5.15347	-0.99298
21	H	-1.52266	-5.17439	-0.83589
22	N	-2.3053	-0.02407	-0.64466
23	C	-3.11809	-1.14648	-0.54156
24	C	-3.13438	1.086677	-0.54284
25	C	-4.51018	-0.72498	-0.36419
26	C	-4.51987	0.646754	-0.36552
27	H	-5.36205	-1.37413	-0.22689
28	H	-5.38063	1.285398	-0.23327
29	C	-2.70212	2.435718	-0.55118
30	C	-3.76157	3.488225	-0.44736
31	C	-3.8223	4.35408	0.67027
32	C	-4.73533	3.653354	-1.45737
33	C	-4.81652	5.32358	0.752185
34	H	-3.11251	4.266363	1.483841
35	C	-5.70847	4.644793	-1.33888
36	H	-4.73394	3.03031	-2.34338
37	H	-4.9024	5.995365	1.596828
38	H	-6.46238	4.804799	-2.09797
39	C	-2.66539	-2.48987	-0.55502
40	C	-3.70965	-3.55718	-0.47379
41	C	-3.73182	-4.47661	0.602251

42	C	-4.7139	-3.68231	-1.46049
43	C	-4.72012	-5.45309	0.670156
44	H	-2.99836	-4.42299	1.397537
45	C	-5.68048	-4.68099	-1.35685
46	H	-4.74145	-3.02126	-2.31801
47	H	-4.77681	-6.16482	1.483806
48	H	-6.45717	-4.80911	-2.09888
49	C	2.252224	-2.45165	-0.88208
50	C	3.309609	-3.50681	-1.00657
51	C	3.608531	-4.35787	0.080141
52	C	4.03913	-3.68946	-2.20439
53	C	4.600948	-5.32897	-0.04183
54	H	3.088056	-4.26233	1.025329
55	C	5.016921	-4.67743	-2.28474
56	H	3.841937	-3.0805	-3.07829
57	H	4.864823	-5.98832	0.774364
58	H	5.591434	-4.85292	-3.18527
59	C	2.212666	2.481044	-0.8939
60	C	3.259385	3.543185	-1.0131
61	C	4.248945	3.700694	-0.01406
62	C	3.300241	4.420392	-2.11988
63	C	5.213711	4.694907	-0.13567
64	H	4.262294	3.06717	0.864335
65	C	4.290417	5.398028	-2.20518
66	H	2.58126	4.338634	-2.92579
67	H	5.979981	4.853536	0.612168
68	H	4.356953	6.079122	-3.04329
69	N	-5.74116	5.461519	-0.24576
70	N	5.226844	5.526762	-1.2208
71	N	5.289749	-5.47836	-1.21102
72	N	-5.67751	-5.54689	-0.30159
73	C	6.289303	6.573037	-1.30895
74	H	6.142944	7.168878	-2.20965
75	H	6.224839	7.217597	-0.43069
76	H	7.263723	6.0824	-1.34426
77	C	-6.78886	6.518996	-0.11515
78	H	-7.44706	6.48786	-0.98308
79	H	-7.36732	6.331127	0.791339
80	H	-6.30619	7.496882	-0.0566
81	C	-6.71127	-6.62013	-0.19589
82	H	-7.19875	-6.55026	0.778177
83	H	-7.4521	-6.48733	-0.98354
84	H	-6.22764	-7.59341	-0.30421
85	C	6.356711	-6.51853	-1.32738
86	H	6.391623	-7.08919	-0.40047
87	H	6.123217	-7.18483	-2.16047

88	H	7.320571	-6.0327	-1.49571
89	Cu	-0.2288	-0.00243	-0.26499
90	O	-0.4073	0.041072	1.731734
91	C	0.455056	0.040803	2.677213
92	C	2.725352	0.008606	3.523094
93	H	2.120998	-0.0062	1.504208
94	C	0.897953	0.06859	5.127538
95	C	2.335433	0.034854	4.832617
96	H	3.765918	-0.01681	3.224903
97	N	0.049223	0.068722	3.98905
98	H	-0.95474	0.090094	4.159615
99	O	0.388305	0.096064	6.277408
100	C	3.3042	0.03136	5.986458
101	H	3.170327	0.923679	6.610329
102	H	3.138356	-0.84009	6.631827
103	H	4.338967	0.008177	5.631053
104	N	1.806046	0.011721	2.473939

Table S8. The Optimized Bond-Lengths and Angles for CuTMPyP4-(κ-*O*-thymine) obtained at the B3LYP/LanL2DZ Level

Atom No	Symbol	Atom 1	Atom 2	Atom 3	Bond	Angle	Dihedral
1	N						
2	C	1			1.396283		
3	C	1	2		1.394766	106.6955	
4	C	2	1	3	1.45972	109.4092	1.581469
5	C	4	2	1	1.374421	107.2048	-1.41101
6	H	4	2	1	1.079674	126.2678	179.0672
7	H	5	4	2	1.079888	126.5718	-177.899
8	N	1	3	5	2.939283	81.2966	-173.452
9	C	8	1	3	1.388534	171.2904	-171.658
10	C	8	1	3	1.390535	81.66613	-2.54895
11	C	9	8	1	1.464218	109.4882	168.9496
12	C	11	9	8	1.371714	107.1047	-0.45605
13	H	11	9	8	1.079775	126.2897	178.5766
14	H	12	11	9	1.079601	126.5812	-179.002
15	N	1	3	5	2.940471	169.9867	-144.779
16	C	15	1	3	1.39061	81.45689	147.3116
17	C	15	1	3	1.388058	171.3574	-18.3866
18	C	16	15	1	1.463387	109.478	-177.179
19	C	18	16	15	1.372236	107.0453	-0.35344
20	H	18	16	15	1.080119	126.1679	177.9572
21	H	19	18	16	1.079906	126.6418	177.7025
22	N	15	1	3	2.942287	90.13393	-27.6242
23	C	22	15	1	1.389624	81.50873	-177.599
24	C	22	15	1	1.38979	170.2207	-27.4787
25	C	23	22	15	1.465282	109.4099	174.7506
26	C	25	23	22	1.371771	107.1119	0.130859
27	H	25	23	22	1.079773	126.2975	-178.101
28	H	26	25	23	1.079935	126.6458	-178.472
29	C	24	22	15	1.416628	125.3541	28.65646
30	C	29	24	22	1.496995	116.9476	179.2398
31	C	30	29	24	1.415096	121.0199	115.7088
32	C	30	29	24	1.412647	121.3619	-64.2529
33	C	31	30	29	1.391086	120.2392	-179.785
34	H	31	30	29	1.083233	121.0319	-0.90102
35	C	32	30	29	1.39427	120.2297	-179.756
36	H	32	30	29	1.083147	121.1129	-0.8512
37	H	33	31	30	1.082632	122.3501	179.2184
38	H	35	32	30	1.081763	122.1384	179.3785
39	C	23	22	15	1.417676	125.3033	-3.05717
40	C	39	23	22	1.495405	116.9253	-178.166
41	C	40	39	23	1.415522	121.0518	-118.947
42	C	40	39	23	1.413416	121.4279	60.68613
43	C	41	40	39	1.390996	120.2788	179.2283

44	H	41	40	39	1.083196	121.022	0.589458
45	C	42	40	39	1.393688	120.284	-179.609
46	H	42	40	39	1.08309	121.1144	1.464979
47	H	43	41	40	1.082497	122.3752	-179.192
48	H	45	42	40	1.081792	122.127	-179.465
49	C	16	15	1	1.415655	125.5132	-0.56114
50	C	49	16	15	1.498973	116.7463	179.7801
51	C	50	49	16	1.412304	120.6361	-68.6359
52	C	50	49	16	1.414335	121.6749	111.2858
53	C	51	50	49	1.393846	120.2123	-179.483
54	H	51	50	49	1.083237	121.1015	-0.46928
55	C	52	50	49	1.392341	120.1815	179.8505
56	H	52	50	49	1.083245	121.1141	-1.11887
57	H	53	51	50	1.081917	122.1563	179.1481
58	H	55	52	50	1.082504	122.3945	179.5576
59	C	10	8	1	1.415996	125.3125	1.190093
60	C	59	10	8	1.495986	117.1435	179.3239
61	C	60	59	10	1.414969	120.8036	117.7283
62	C	60	59	10	1.412842	121.5659	-62.38
63	C	61	60	59	1.390692	120.1984	-179.808
64	H	61	60	59	1.083097	121.0723	-0.90959
65	C	62	60	59	1.394093	120.2567	-179.808
66	H	62	60	59	1.083096	121.1193	-1.00536
67	H	63	61	60	1.082405	122.3754	179.3254
68	H	65	62	60	1.082009	122.1745	179.3575
69	N	35	32	30	1.364921	120.6815	-0.46061
70	N	65	62	60	1.364725	120.6223	-0.37749
71	N	53	51	50	1.3652	120.6914	-0.46678
72	N	45	42	40	1.365054	120.6994	0.338674
73	C	70	65	62	1.493747	120.7676	179.9941
74	H	73	70	65	1.089823	109.6455	2.087007
75	H	73	70	65	1.091304	108.9169	121.9239
76	H	73	70	65	1.091543	108.7852	-118.013
77	C	69	35	32	1.494322	120.6794	-179.65
78	H	77	69	35	1.089719	109.4904	0.428798
79	H	77	69	35	1.091622	108.8034	120.2626
80	H	77	69	35	1.092089	109.1761	-119.544
81	C	72	45	42	1.493873	120.592	-179.739
82	H	81	72	45	1.091474	109.0395	-126.35
83	H	81	72	45	1.089432	109.3807	-6.63884
84	H	81	72	45	1.092206	109.0727	113.4124
85	C	71	53	51	1.494621	120.1989	179.6783
86	H	85	71	53	1.089055	108.7414	3.402657
87	H	85	71	53	1.092028	109.3529	123.1196
88	H	85	71	53	1.092423	109.4115	-116.309
89	Cu	22	15	1	2.111031	45.94712	-16.389

90	O	89	22	15	2.00516	95.26452	-101.484
91	C	90	89	22	1.279681	132.5116	178.3683
92	C	91	90	89	2.422972	152.7925	0.055491
93	H	91	90	89	2.038017	97.21596	0.366969
94	C	92	91	90	2.432535	61.70839	-179.74
95	C	92	91	90	1.366593	93.83775	-179.699
96	H	92	91	90	1.082747	143.5766	0.221515
97	N	91	90	89	1.373461	120.4353	-179.661
98	H	97	91	90	1.018576	116.8471	-0.05967
99	O	94	92	91	1.258053	155.1932	-179.97
100	C	95	92	91	1.506609	123.3917	179.9523
101	H	100	95	92	1.096982	110.8054	120.8242
102	H	100	95	92	1.097012	110.8046	-120.5
103	H	100	95	92	1.094346	111.0591	0.156198
104	N	91	90	89	1.366507	123.8022	0.237992
